# Quality and Multifunctionality in Mobile Apps for Gestational Diabetes: Systematic App Review

**DOI:** 10.2196/76862

**Published:** 2026-02-05

**Authors:** Qimeng Zhao, Alison Cooke, Lishan Huang, Yimin Tang, Dawn Dowding

**Affiliations:** 1 Division of Nursing, Midwifery and Social Work, Faculty of Biology, Medicine and Health, University of Manchester, Room 6.306, Jean McFarlane Building, Oxford Road, Manchester, M13 9PL, United Kingdom, 44 7410902025; 2 Centre for Research & Education Excellence (CeNREE), Royal Stoke University Hospital, University Hospitals of North Midlands NHS Trust, Stoke on Trent, United Kingdom; 3 School of Nursing and Midwifery, Keele University, Keele, United Kingdom

**Keywords:** diabetes, gestational, pregnant woman, smartphone, mobile health, mHealth, app, behaviour change, mobile phone

## Abstract

**Background:**

The use of mobile health (mHealth) apps can assist with the management of gestational diabetes (GDM). Although a number of studies have demonstrated their efficacy in improving maternal-fetal outcomes, opinions differ regarding their usability and overall quality. Poorly designed apps, with ill-conceived features or inappropriate content, may pose a threat to patient safety. Nevertheless, very few studies provide in-depth evaluations of app design quality, and the diversity of features and techniques used remains insufficiently explored.

**Objective:**

We aimed to evaluate the quality and multifunctionality of commercially available mHealth apps for GDM.

**Methods:**

This is a systematic app review guided by the TECH (target user, evaluation focus, connectedness, and health domain) framework and the PRISMA (Preferred Reporting Items for Systematic Reviews and Meta-Analyses) 2020 checklist. Searches were conducted on the Apple App Store and Google Play. Apps were screened by name, description, and full navigation to identify inclusions. The quality of the apps was evaluated using the Mobile App Rating Scale and IMS Institute for Healthcare Informatics Functionality Score. Multifunctionality of the apps was evaluated using the GDM-adapted features and techniques list developed from the App Behavior Change Scale, NICE (National Institute for Health and Care Excellence) 2015 guidelines, and previous studies. The general features list, which contains instruction, data security, customization, and technical issues, was derived from previous studies.

**Results:**

The search (June 2024) identified 23 commercially available apps from UK app stores. The overall app quality was evaluated to be satisfactory (Mobile App Rating Scale: mean 4.0, SD 0.36; IMS Institute for Healthcare Informatics Functionality Score: mean 5.83, SD 3.03). The multifunctionality evaluation found that the apps had a mean of 17.95 and SD of 7.31 across all 45 items. Overall, our findings suggested that mHealth apps for GDM achieved a certain level of multifunctionality. However, their feature types and supporting digital techniques are relatively basic. The apps focused on education and managing blood glucose control rather than integrating other self-monitoring data and pregnancy-relevant management into their design. The digital techniques used to achieve these features included text and manual operation, rather than other automated features.

**Conclusions:**

This is the first app review to consider the relationship between app features and usability for women with GDM. Future app development should integrate a wide range of pregnancy-relevant information and more automated features and use advanced digital techniques to enable a holistic digital solution for women with GDM.

## Introduction

### Challenges in Gestational Diabetes Self-Management

The self-management of gestational diabetes (GDM) usually includes a series of complex lifestyle behavioral changes such as diet, exercise, and continuous self-testing of blood glucose [1]. This systematic approach aims to help women maintain optimal blood glucose levels and minimize complications for both mother and baby. However, previous research highlights the challenges for women with GDM, who find it hard to understand or follow the instructions from doctors [2]. Women have to make decisions on food choices daily, and this is done through effective cooking strategies [3]. It also challenges women to understand the labels and nutritional values of food [4]. Guidelines across countries emphasize the importance of strengthening physical activities and regard aerobic activities as acceptable approaches [5]. However, this requires carefully choosing the level of intensity and the types of exercise [5].

### Mobile Health Apps Help With Self-Management of GDM

GDM digital technologies have covered various areas of GDM management, providing features including aspects of educational information, health behavior coaching, data recording, and communication interfaces for women [6]. By analyzing the overall effectiveness, previous literature found that GDM digital technologies could provide comparable quality of care to face-to-face visits with health care professionals or midwives, manifesting in heightened maternal health conditions and increased rates of natural childbirth, alongside diminished occurrences of adverse maternal and neonatal outcomes [7-9].

There are various forms of digital technologies, such as mobile apps, webpages, and digital devices, among which mobile apps are one of the most common types of technology used [9]. The rise in global smartphone penetration has led to the evolution of mobile apps, which are now one of the most representative forms of GDM digital technologies [8-10]. Mobile phones possess advantages in terms of accessibility as they are portable devices, thereby facilitating women’s access to digital services irrespective of their geographical locations [11,12].

### Variation in Perceived Usability of Mobile Health Apps for GDM

However, unlike the overall optimistic results from the evaluation of effectiveness, our previous review and other studies found a variation in pregnant women’s views on the app’s usability [13,14]. Women had positive views of apps when they supported self-monitoring and viewed them as beneficial tools for obtaining information about GDM, making informed dietary decisions, and facilitating exercise regimens [13,15-17]. The technologies also led to increased levels of adherence, concentration, and satisfaction among women [7,14]. The apps offered platforms for the women and clinicians to keep continuous interaction, fostering the client-clinician relationship [16,18]. This mitigated various concerns that women encounter in managing their condition, demonstrating efficacy in facilitating women’s self-management practices [7-9]. In the meantime, apps added to women’s emotional burden with problematic features and content [13]. For example, redundant processes and frequent failures in data recording reduced women’s motivation for using the technology [18,19]. The differences between the information provided by apps and clinicians confused the women [16]. Evidence suggests that mobile health (mHealth) apps might unintentionally lead to increased unhealthy behavior [20]. mHealth apps designed inappropriately may contain inaccurate or incomplete information that misleads users or provide insufficient support for managing high-risk behaviors, which could affect users’ safety [21].

### Challenges to Conducting a Comprehensive Evaluation of GDM Apps

To ensure that women are offered apps of the best quality, health care practices face challenges in evaluating and selecting suitable options. The current era is facing the results of unregulated mHealth markets, which flourish in the number of emerging mHealth apps but vary in quality [22]. The increasing study of app quality evaluation has triggered a discussion on the definition of app quality. Studies found that the criteria used for evaluating app quality were heterogeneous [23,24]. In addition, established criteria lack precise definitions, which hinders the assessment of app quality.

Two previous reviews have evaluated the functionality and usability of GDM mHealth apps [6,25]. Both reviews primarily relied on a single quality criterion: the Mobile App Rating Scale (MARS). However, neither study addressed how well app features are adapted to GDM self-management content. Similarly, they offered no practical insights to assist health care professionals in selecting apps that are both functional and user-friendly. What is missing from the current literature is an evaluation of how these apps contain features suitable for GDM care, and guidance that helps health care professionals when selecting usable apps.

### Objectives

This study reviewed commercially available mobile apps to explore app quality and multifunctionality, targeted at antenatal care for women with GDM or pre-existing diabetes during pregnancy, available in the UK app market, and in the English language. This systematic evaluation only includes free-download apps and analyzes the available free-of-charge features, providing insights into the types of features that are most accessible for women with GDM. We assessed the quality of the apps using standardized evaluation scales. We evaluated the app’s multifunctionality based on app features and content tailored specifically to the GDM population, as well as general features that affect the normal use of apps.

## Methods

### Study Design

This study followed the TECH (target user, evaluation focus, connectedness, and health domain) framework proposed by Gasteiger et al [26] (2023), which is tailored for the context of reviewing and evaluating the quality of commercially available mHealth apps as opposed to the systematic literature review methodology. Informed by the traditional systematic review process, the TECH framework aims to help conduct reviews on mobile apps by establishing search strategies, the use of eligibility criteria, and a comprehensive quality appraisal. It provides guidance to conduct a search, and has been applied in recent studies to evaluate mHealth apps, including risk assessment and self-management tools, by examining both their functionality and content [27-29].

**Figure 1.**
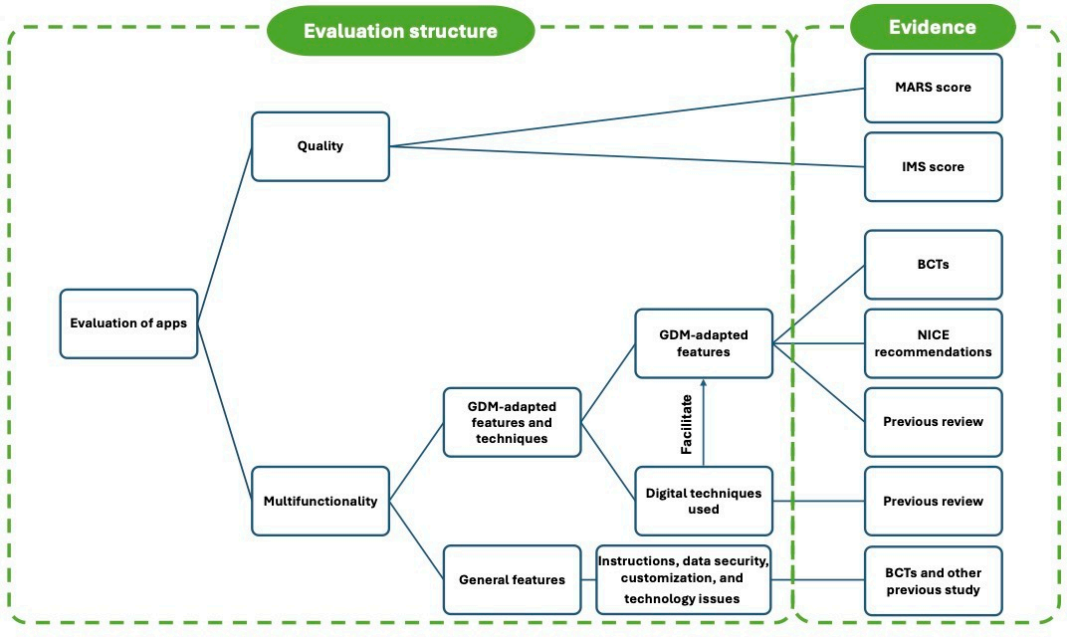
Evaluation structure. BCT: behavior change technique; GDM: gestational diabetes; IMS: The IMS Institute for Healthcare Informatics Functionality Score; NICE: National Institute for Health and Care Excellence; MARS: Mobile App Rating Scale.

This study consists of 2 major domains of evaluation: app quality and app multifunctionality. Figure 1 shows the structure of the evaluation elements and the evidence supporting the evaluation. The app quality evaluation in this study aimed to explore the type of features being used in the included apps and used generalized tools to quantify the level of quality. The measurement was informed by the TECH framework and guidance by introducing both the MARS and the IMS Institute for Healthcare Informatics Functionality Score (IMS). The aggregation of the 2 quality appraisal tools helps to evaluate mobile apps more comprehensively.

The multifunctionality evaluation aimed to conduct a GDM context-tailored assessment on the behavior change techniques (BCT) used in the apps, as well as other essential features needed to be enabled for normal app usage. Therefore, there were two elements in the multifunctionality evaluation: (1) the GDM-adapted features and techniques, which refer to the app features aligned with BCTs, along with the digital techniques used to enable the features; and (2) the general features, which refers to the essential features that enable the normal usage of apps, including instructions, data security, customization, and technical issues.

### Eligibility Criteria

Following the guidance of the TECH framework, a scoping search on Apple App Store and Google Play in the UK context was run in March 2024 to identify eligibility criteria [26]. This study aims to identify apps that include GDM as one of the targeted user groups and that eligible apps must refer to GDM in their description to qualify for inclusion in this study. From this process, three potential types of mobile apps were identified: (1) mobile apps for GDM management, (2) mobile apps for pregnancy management with additional features for blood glucose control, and (3) mobile apps tailored for diabetes patients but claimed to be suitable for women with GDM. Additionally, the mobile apps were available in the UK context and the English language, free to download, running on Android (Google LLC) or iOS (Apple Inc). The eligibility criteria are listed as follows (Multimedia Appendix 1):

1. Target user (T): mobile apps tailored for GDM, pregnancy, and diabetes users, with GDM as one of the targeted user groups.
2. Evaluation focus (E): content of information, quality, and functionality of the mobile apps that are suitable for users with GDM.
3. Connectedness (C): mobile apps with external devices such as glucose meters and smartwatches.
4. Health domain (H): mobile apps suitable for antenatal behavioral change for women with GDM rather than prevention or postpartum care.

### Search

The search keywords "gestational diabetes," "GDM," and "pregnancy diabetes" were applied in the search strategy. The final search was conducted by 3 reviewers on the Apple App Store and Google Play in June 2024. Due to the Apple App Store’s online page design, it was not possible to export the search results into screening software to remove duplicates. Additionally, the mobile app search results were subject to frequent changes over time. To avoid unnecessary duplication in the search, all 3 reviewers searched and performed screening on the same day.

### Screening

The screening process was guided by the PRISMA (Preferred Reporting Items for Systematic Reviews and Meta-Analyses) checklist and the TECH methodology [26,30]. Two steps of screening were applied, including title screening and full mobile app screening. The mobile app name and description screening process was conducted on the search results pages to identify potential mobile apps for inclusion. We contacted the app companies to access those apps with limited access during the retrieval process. A subsequent full mobile app screening was conducted to check the relevance and feature operability. To include the mobile apps which were usable at the technological level as well as suitable for the targeted population, the full mobile app screening process made a decision of inclusion when (1) the screened mobile app was available in English; (2) no constant technical glitch or crash occurred when operating; and/or (3) the mobile app mentioned GDM at least once in options, information, or features. A snowballing strategy was used after the full mobile app screening process to check the relevant-recommended apps generated within the search results pages of the final-included mobile apps, such as the “you might also like” and “similar apps” pages, to reduce missing mobile apps.

During the screening process, 2 pairs of authors independently screened the search results in Google Play and Apple App Store. The level of agreement between the reviewers was determined using Cohen κ to calculate interrater reliability. To minimize the potential for duplication inherent in app store searches, the analysis was based on the results from a single search term. Discrepancies between the paired authors were discussed and resolved. The results of eligible mobile apps were checked and agreed upon by the other authors. The devices used to search and screen the apps were iPhone 11 Pro Max (iOS 17.5), iPhone 11 (iOS 17.5), iPhone X (iOS 16.7.8), Samsung Galaxy J5 (Android v8.1 Oreo), and Huawei Mate 10 (HarmonyOS 4.0).

### Quality Appraisal

#### Overview

Two quality appraisal tools, which are widely applied for mHealth evaluation, the MARS [26,31] and the IMS tool [32], were used to evaluate the mHealth apps. The MARS (26 items) was used to evaluate the technical design, and the IMS (11 items) to evaluate the number of functions.

The MARS was scored from inadequate to excellent with a corresponding score from 1 to 5 in the 5-point Likert scale. This review followed the recommendation of MARS to calculate MARS scores using the 16 items of app quality rating. The subjective quality and perceived impact items were combined as subjective quality (10 items) for evaluation in this review. The IMS [32] items were coded as 1 per item if app features were presented and otherwise 0 per item, generating a functionality score ranging from 0 to 11 for each app. The overall mapping scores representing the frequencies of applied features were presented in a radar graph as recommended [32].

Quality appraisal was conducted by 2 pairs of reviewers. The agreement rate between the reviewers using different scales was calculated, with interclass correlation coefficients (ICCs) for MARS results [33] and Cohen κ for the IMS results. The level of agreement in ICCs, which ranges between 0 (no agreement) and 1 (perfect agreement), was reported in the value and followed the level categorization as either poor (<0.40), fair (0.40‐0.59), good (0.60‐0.74), or excellent (0.75‐1.0) [34]. The calculation was conducted using IBM SPSS (version 29; IBM Corp).

#### Data Extraction

Data extraction focused on two categories: (1) app characteristics, which included basic information such as the app operating system, version, and size; and (2) multifunctionality, which included GDM-adapted features and techniques, as well as general features. A list of uncategorized app features and techniques was initially extracted and used to inform the GDM-adapted features and techniques list and the general features list. The two lists contributed to the evaluation of the app’s multifunctionality (Multimedia Appendix 2). After the development of the evaluation lists, the apps were reviewed again by 1 author and extracted for the items on the lists. The extracted features and techniques of individual apps were checked and discussed to reach an agreement in the author group.

The GDM-adapted features and techniques lists included types of app features (GDM-adapted features) and digital techniques (GDM-adapted techniques). GDM-adapted features refer to the app feature content, which served as a digital solution to clinical interventions for GDM management. For example, blood glucose data recording served as a digital solution to a paper diary for blood glucose documentation. GDM-adapted techniques are the digital techniques used to enable the app features. For example, Bluetooth (Bluetooth Special Interest Group) techniques and manual recording are both digital techniques used to enable the data recording app feature. General features referred to instructions, data security, and customization, which were extracted based on other studies [35,36].

The development of the GDM-adapted features and techniques list included app features aligned with BCTs [36], NICE (National Institute for Health and Care Excellence) 2015 recommendations [37], and our previous review on women’s preferences [13]. BCTs include strategies such as education, goal setting, self-monitoring, feedback, and reinforcement that encourage healthy behaviors [36]. The 2015 NICE guideline recommends managing GDM through individualized diet and exercise, along with blood glucose monitoring [37]. It also includes pregnancy management guidance by including frequent antenatal visits and monitoring of fetal growth and well-being [37]. Our previous review found that women with GDM valued digital tools with education, personalized guidance, and easy data management [13]. It highlights the need for more usable technologies that support self-monitoring of GDM [13]. Integrating these sources created a clear framework for assessing app features, making sure the GDM-adapted features and techniques list reflects behavior change, clinical care, and women’s needs when managing GDM. Additionally, some basic digital techniques, such as text-based educational information and data recording approaches, were added to the final list of app features without direct indications from literature or recommendations.

### Data Analysis and Presentation

The extracted features and techniques of apps were analyzed and presented in aggregated data. Categorical data, including app characteristics items, GDM-adapted features, GDM-adapted techniques, and general features, were summarized as frequencies and percentages. Continuous data, including app size, MARS score, and IMS score, were presented as means with SDs. App characteristics, MARS score, GDM-adapted features and techniques, and general features were summarized in tables to provide a clear presentation of results. The IMS score was presented in a radar chart to illustrate the distribution of IMS functionalities across 10 domains.

### Ethical Considerations

This study did not involve human participants, animals, or any collection of personally identifiable information. All data analyzed were publicly available in the Apple App Store and Google Play Store. Accordingly, institutional review board approval was not required. As no human subjects were enrolled, informed consent was not applicable.

## Results

### Search Results

The search identified 1066 apps, 330 from the Apple App Store and 736 from Google Play. Two paired authors independently screened the search results in both app stores, the Apple App Store, resulting in 28 eligible mobile apps from the Apple App Store, and 21 in Google Play. The interrater reliability in both pairs indicates a minimum of substantial agreement, with Cohen κ values of 0.62 and 0.91. The title and description screening process resulted in 49 potentially eligible mobile apps in the two app stores. The mobile apps available in both operating systems were not combined at this step.

The 49 eligible mobile apps were retrieved and installed before the full mobile app screening process. This process led to 10 exclusions due to activation (n=7), subscription (n=1), and technical crash issues (n=2). The retrieving, installing, and checking process led to 39 (n=21 in iOS, n=18 in Android) inclusions for the full mobile app screening. The full app screening led to 10 exclusions (n=5 in iOS, n=5 in Android). The reasons for exclusion included irrelevance (n=9) and language (n=1). Perfect agreement was identified between the 2 pairs of authors. Mobile apps available in both operating systems (n=8) were then combined in number, resulting in 21 eligible mobile apps.

**Figure 2.**
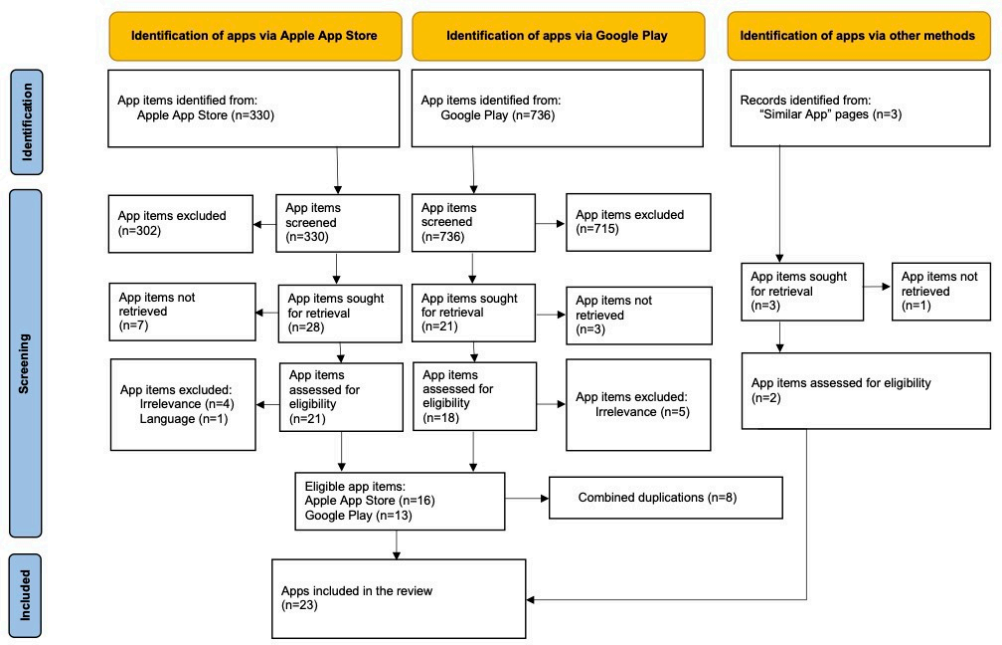
PRISMA diagram. PRISMA: Preferred Reporting Items for Systematic Reviews and Meta-Analyses.

A subsequent snowballing process generated the inclusion of 2 mobile apps (available in both operating systems). The search and screening process contributed to the eventual inclusion of 23 eligible mobile apps for this study. Figure 2 illustrates the PRISMA diagram for the screening process (Multimedia Appendix 3 for the enlarged PRISMA diagram).

### App Characteristics

Of the 23 reviewed apps, 56% (n=13) were adapted to both operating systems (iOS and Android), followed by 22% (n=5) of the apps which were exclusively for iOS, and 22% (n=5) of the apps which were exclusively for Android. Most (n=16, 70%) of the apps were updated in 2024. On average, the apps were of mean 66.43 and SD 53.46 MB, ranging between 6.2 and 223.4 MB. All the apps were free to download, with 48% (n=11) free to access all features, and 52% (n=12) required a subscription. The major targeted user groups of the apps were people with diabetes (n=12, 52%), women with GDM (n=7, 30%), followed by women who are pregnant (n=3, 13%) and women with diabetes who are pregnant (n=1, 4%).

We categorized the apps into 4 major types based on the characteristics of features: multipurpose, self-monitoring, educational, and communicational. Self-monitoring apps were identified by their ability to assist women’s behavior for self-monitoring, such as recording data and reminding them of self-tests, with a total of 5 apps. Educational apps were identified by their ability to offer women information, with a total of 5 apps. Communicational apps were identified by their ability to provide a platform for women to communicate with their peers and health care professionals, with a total of 2 apps. The apps that used more than one of the mentioned characteristics of features were identified as multipurpose apps (n=11). All apps (n=23) were free to download, with all apps that were tailored for GDM or pregnant users (n=11) being free to access all features; meanwhile, the other 12 apps for general diabetes users needed to subscribe for full access. Table 1 shows the characteristics of the reviewed apps.

**Table 1.** App characteristics.

Evaluations	Values	
Operating systems, n (%)		
Both	13 (56)	
iOS	5 (22)	
Android	5 (22)	
Last update, n (%)		
2024	16 (70)	
2023	4 (17)	
2022 and before	3 (13)	
Size (MB), n (%)		
0‐50	11 (48)	
50‐100	7 (30)	
>100	5 (22)	
Targeted population, n (%)		
Diabetes	12 (52)	
GDM^a^	7 (30)	
Pregnancy	3 (13)	
Diabetes in pregnancy	1 (4)	
App type, n (%)		
Multipurpose	11 (48)	
Educational	5 (22)	
Self-monitoring	5 (22)	
Communicational	2 (9)	
Price, n (%)		
Subscription for full access	12 (52)	
Free full access	11 (48)	
MARS^b^ quality score, mean (SD)	4.0 (0.37)	
IMS^c^ score, mean (SD)	5.83 (3.10)	

a GDM: gestational diabetes.

b MARS: Mobile App Rating Scale.

c IMS: The IMS Institute for Healthcare Informatics Functionality Score.

### Quality Appraisal

#### Overview

Overall, the mean MARS score was 4.0 and SD 0.36, and the mean IMS score was 5.83 and SD 3.03 (see details in Multimedia Appendix 4). The mean MARS quality score indicates a satisfactory level of quality, which appears to be higher than the average MARS quality score reported for mHealth apps in the literature (mean 4.0, SD 0.36 vs mean 3.51, SD 0.71) [38]. Regarding individual MARS quality score domains, the MARS engagement domain, which is relevant to coaching and personalization features, scored the lowest (mean 3.56, SD 0.60) compared to other MARS domains (MARS functionality: mean 4.48, SD 0.72, MARS esthetics: mean 4.12, SD 0.48, MARS information: mean 4.02, SD 0.44). Apps were also evaluated based on their different types of functionality using the MARS quality score: the multipurpose apps, educational apps, self-monitoring apps, and communicational apps. Multipurpose (mean 4.06, SD 0.44) and self-monitoring (mean 4.02, SD 0.25) apps appeared to score higher than other types of apps (educational apps: mean 3.92, SD 0.37, communicational apps: mean 3.84, SD 0.47).

**Figure 3.**
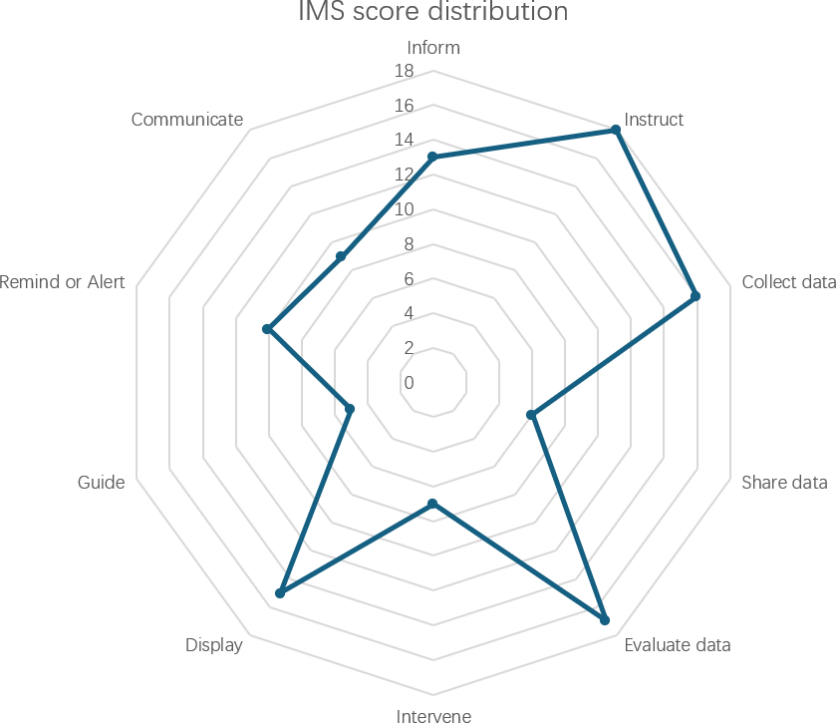
Distribution of features by IMS scores. IMS: The IMS Institute for Healthcare Informatics Functionality Score.

The mean IMS score indicated a moderate level of the number of app features. Multipurpose (mean 7.91, SD 2.12) and self-monitoring (mean 6.60, SD 1.14) apps tended to score higher than the other two types of apps. Figure 3 shows the radar graph of the distribution of features scored by the IMS. Among all the 10 IMS features, instructions (n=18), data management features (n=18), and information (n=13) were the predominant features used by the 23 reviewed apps. The guide feature scored lowest compared to other IMS features (n=5).

Reviewers had substantial agreement on their evaluation of iOS apps; IMS (κ=0.834, 95% CI 0.74-0.93, *P*<.001) and MARS (ICC=0.808, 95% CI 0.742‐0.858) scores. A similar level of agreement was observed for Android apps, with IMS (κ=0.851, 95% CI 0.76-0.95, *P*<.001) and MARS (ICC=0.755, 95% CI 0.67‐0.82).

#### Multifunctionality

The analysis of features and techniques was based on the exploration of features that would need to be integrated into an app to assist specifically with GDM. We incorporated the BCTs [36], NICE recommendations [37], and our previous review on women’s preferred features [13]. The process generated a list of GDM-adapted features and techniques (Table 2, details in Multimedia Appendix 5). The features were categorized into 5 domains: education, data management, coaching features, communication features, and pregnancy management.

**Table 2.** GDM^a^-adapted features and techniques (N=23).

GDM-adapted features and techniques	Evidence^b^	Frequencies of applied features
Education (n=18)		
Educational content features		
Knowledge about GDM diagnosis	N, P	10
Treatment types for GDM	N	7
Low GI^c^ index food choices	B, N	15
Food recipes	B	4
Evidence-based information	B, P	11
Self-monitoring skill	B, N, P	8
Educational information delivery techniques		
Text	—^d^	18
Pictures or graphs	P	9
Videos	P	4
Data management (n=16)		
Data management content features		
Blood glucose	N, P	14
Food	N, P	8
Exercise	N, P	7
Abnormal symptom reporting	N	4
Medication	N	6
Data recording techniques		
Manual (number or text)	—	14
Automatic glucose data transmission	P	4
Automatic exercise data transmission	P	3
Food or exercise database	P	7
Image recognition techniques	P	3
Data visualization techniques		
Tables with text	—	11
Graphs	P	15
Combination charts	P	3
Other data management features		
Digital feedback	B, N, P	14
Data export	B	8
Personal-tailored information or suggestions	B, N, P	3
Coaching features (n=11)		
Reminders	B, P	9
Compliance motivator	B, P	5
Rewarding for milestone achievements	B	3
Communication features (n=9)		
Communication with HCPs^e^	B, N, P	6
Communication with peers	B, P	6
Pregnancy management features (n=5)		
Pregnancy progression education and management	N, P	4
Fetal monitoring	N, P	3

a GDM: gestational diabetes.

b Evidence: B: behavior change techniques; N: NICE (National Institute for Health and Care Excellence) recommendations; P: women’s preferred features.

c GI: glycemic index.

d Not applicable.

e HCP: health care professional.

Table 3 summarizes the predominant GDM-adapted features and techniques applied by the reviewed apps. The analysis reveals a strong emphasis on educational content (n=18) and data management (n=16) within the mobile apps. The educational components of the apps focus on delivering content critical for GDM management, mainly including knowledge about GDM diagnosis (n=10) and low GI index food choices (n=15). Data management predominantly focuses on collecting blood glucose data, with over 50% (n=14) of apps, rather than food (n=8) and exercise tracking (n=7).

**Table 3.** Predominant applied GDM^a^-adapted features and techniques.

App names	Predominant educational features		Predominant data management features	Predominant techniques		
	GDM diagnosis	Food choices	Blood glucose	Text information delivery	Manual data recording	Text data visualization
GDm-Health	✓	✓	✓	✓	✓	✓
Malama	✓	✓	✓	✓	✓	✓
MyManis	✓	✓	✓	✓	✓	✓
Flora	✓	✓		✓		
hug + u			✓	✓	✓	✓
myFetalLife			✓	✓		
Blood Sugar - Diabetes App		✓	✓	✓	✓	✓
Blood Sugar Tracker - Diabetes	✓		✓	✓	✓	✓
Diabetes:M	✓	✓	✓	✓	✓	✓
DiabTrend		✓	✓	✓	✓	
MyNetDiary			✓	✓	✓	✓
Gestational Diabetes Diet		✓		✓		
glukoGest	✓			✓		
Pregnant with diabetes	✓	✓		✓		
NewToType2	✓	✓		✓		
Pingoo	✓	✓		✓		
Gestational Diabetes Tracker			✓		✓	✓
Carbs & Cals		✓				✓
Diabetic diary-Glucose tracker			✓		✓	✓
forDiabetes			✓		✓	✓
mySugr			✓		✓	
Hlgedi		✓		✓		
Diabetes Forum		✓		✓		

a GDM: gestational diabetes.

Text-based educational information was nearly ubiquitous, and core tracking features—particularly for blood glucose—were widely implemented. Text-based information delivery was the most common mode, used in over 50% (n=18) of apps. Manual data recording was predominant (used in 14 apps), and 12 used tables (text) to visualize the data. Among all 23 apps, Malama (a multifunctional, GDM-focused app) and Diabetes:M (a multifunctional, diabetes-focused app) cover all the predominant features and techniques.

We identified the features and techniques applied by fewer than 4 apps as the least applied features. The least addressed features included the provision of food recipes (n=4), abnormal symptom reporting (n=4), rewarding for milestone achievements (n=3), and pregnancy progression and education management (n=4). The least addressed techniques included video information delivery (n=4), automatic blood glucose data transmission (n=4), automatic exercise data transmission (n=3), image recognition techniques (n=3), combination charts (n=3), and personal-tailored information or suggestions (n=3).

### General Features

Overall, most reviewed apps used general features which ensured normal app usage, with 23 apps addressing data security, 22 apps addressing instructions, and 19 apps addressing personalization. There were 5 apps identified as having technical issues that affect normal use, or distractions that disrupt user interactions. General features and technical issues are summarized in Multimedia Appendix 6.

Some general features were applied universally by the apps. These include consenting to privacy policies (n=23) and asking permission to access data (n=23), providing a quick step-by-step introduction (n=16) when users launch the apps. For personalization, collecting users’ demographic data (n=16), asking their preferences for measurement units (n=14), and enabling customized features (n=16), such as reminder timing, were used. Very few apps mentioned the location of data storage (n=5) and the techniques (n=4) used to protect user data.

Additionally, of the 23 evaluated apps, there were 7 with issues that could affect normal user experience, including 5 multipurpose apps (MyManis, hug + u, myFetalLife, Blood Sugar - Diabetes App, and Diabetes:M) and 2 self-monitoring apps (Carbs & Cals and Diabetic diary-Glucose tracker). There were three major issues among these apps: (1) frequent technical glitches, (2) data management issues, and (3) frequent advertisements. Data management issues existed in 4 multipurpose apps, with frequent failure or errors of data recording (MyManis and Diabetes:M), limitations in the data recording feature when inputting decimals and time points (hug + u), and failure of displaying the recorded data in 1 app (myFetalLife). Frequent advertisements took place in 3 apps (Blood Sugar - Diabetes App, Carbs & Cals, and Diabetic diary-Glucose tracker), with advertisements showing up frequently when moving between screens or inputting data, leading to a sense of distraction. Technical glitches and crashes happened to 1 multipurpose app (myFetalLife). While this app contained various features suitable for the management of GDM, technical issues took place frequently and caused crashes and failures when recording the data.

### Usable Apps

This review evaluated the app functionality based on the generated lists of GDM-adapted features and techniques, as well as general functionality, collectively with 45 evaluation items on features and content. On average, the apps applied a mean of 17.95 and SD of 7.31 of all 45 items across domains. For GDM-adapted features and techniques, the apps applied a mean of 10.82 and SD of 5.31 of the 32 items. For general functionality evaluation, the apps applied a mean of 7.13 and SD of 2.58 of the 13 items.

We considered apps usable when they contained features and content higher than the average value in each domain and the total average value, with no distractions or technical issues (Table 4). These 6 apps were either categorized in the multipurpose (n=4) or the self-monitoring (n=2) app type in this review.

**Table 4.** Usable apps and evaluation.

	App type	Health care domain	MARS^a^ quality	IMS^b^ score	Multifunctionality and content	General functionality
Full score or number of features	N/A^c^	N/A	5	11	32	13
Average score or addressed features, mean (SD)	N/A	N/A	4.0 (0.36)	5.83 (3.03)	10.82 (5.31)	7.13 (2.58)
Malama	Multipurpose	GDM^d^	4.63	9	24	9
GDm-Health	Multipurpose	GDM	4.47	8	13	11
MyNetDiary	Multipurpose	Diabetes	4.40	10	16	12
DiabTrend	Multipurpose	Diabetes	4.03	9	20	9
MySugr	SMBG^e^ app	Diabetes	4.13	8	14	11
forDiabetes	SMBG app	Diabetes	4.3	6	11	9

a MARS: Mobile App Rating Scale.

b IMS: The IMS Institute for Healthcare Informatics Functionality Score.

c N/A: not applicable.

d GDM: gestational diabetes.

e SMBG: self-monitoring of blood glucose.

## Discussion

### Overview

This study aimed to provide a comprehensive evaluation of the quality and multifunctionality of commercially available apps on their cash-free features for GDM self-management. By evaluating app quality using the MARS quality and IMS scales, the results showed that the overall app quality is at a satisfactory level. However, regarding individual MARS domains, the MARS engagement domain, which contains coaching and personalized content, was scored lowest among other domains. Following this, the multifunctionality of apps was evaluated by two elements: (1) GDM-adapted features and techniques, namely the app features that fitted into GDM management context, and the corresponding digital techniques used to enable the features, explored the variety of features used by the reviewed apps; (2) general features, which included instruction, data security, customization, and technical issues, explored the app features regarded as essential for basic app usage.

### Primary Findings: Basic Features, Limited Content, and the Need for Advanced Design

The reviewed apps predominantly focused on GDM educational content and blood glucose data recording. In addition, the digital techniques used to enable such features were relatively basic. Most apps relied on text-based approaches for content presentation and required manual operation for data management. Moreover, their content reflected only the most fundamental requirement for GDM, focusing primarily on blood glucose management rather than addressing the broader pregnancy experience.

One of our primary findings is the narrow focus on blood glucose monitoring in the design of many apps. Many of the apps included in this review offered features that could support blood glucose self-management, such as educational resources on self-test skill education and dietary guidance, as well as data recording, digital feedback, and graphic visualization. These features were also identified in our previous study as being preferred by women with GDM, as they facilitated self-management [13]. For example, having educational information in a single app improved easy access and helped women recall information [13]. Initial digital feedback using colored labels, combined with graphic visualization, helped the women interpret their data and adjust their diet according to blood glucose trends [13]. However, most apps lacked a multidimensional approach to GDM management. Even though diet and physical activity are both essential for stabilizing blood glucose levels, very few apps in this review included relevant content and features.

Our findings suggest that features relevant to pregnancy progression management were lacking in available apps. In this study, only a small number of apps provided information or features to support pregnancy management and fetal monitoring. There were only 5 apps that included features or information relevant to pregnancy, such as informing about baby growth over time. The features and information about pregnancy and fetal well-being were more likely to be available in those apps designed for pregnancy care. However, the functionality of these pregnancy-tailored apps in blood sugar control was relatively underdeveloped, typically due to technical issues related to data recording and display. These findings indicated an imbalance in focus in app design, with limited integration of broader pregnancy-relevant needs.

In addition, automated features, including automated data transmission and app-generated behavior change suggestions, were used by only a few apps. Similar patterns are also observed in other mHealth apps for pregnancy care, where basic features and techniques still dominate the feature composition [39-41]. These apps tended to focus on either collecting users’ personal and physiological data or providing normative and comprehensive information, rather than proactively facilitating women’s behavior changes [39-41]. Automated features, such as rewarding on milestone achievements, identifying barriers for behavior change, or providing feedback for behavioral adjustment, are rarely incorporated into the design of pregnancy-related apps [39,40]. Instead, these tasks are sometimes managed through interactions with health care professionals and handled manually [41].

This indicates that the apps currently available and free to download appear to lag behind the evolving needs of women and health care professionals. Women seek tailored guidance for managing their blood glucose, which enhances women’s self-awareness and supports their autonomy in self-management [13]. This is usually achieved through effective communication with health care professionals, where the women can receive one-to-one advice and personalized feedback on their blood glucose levels, diet, and lifestyle adjustment [42,43]. However, limited opportunities for antenatal consultations and the heavy workload of health care professionals have often resulted in limited advice on continuous behavior changes [42,43].

### Implications for Designing Guidelines and Recommendations

Our findings highlight a gap between the potential of technology and its real-world application. Frontier automated techniques are advancing at an astonishing pace, such as deep learning, decision trees, and reinforcement learning, which have been explored for predicting blood glucose levels and providing personalized treatment recommendations [44,45]. These tools can continuously process data such as blood glucose levels, ketonuria, and dietary adherence, identifying patterns and making real-time adjustments [44]. However, our review revealed that many frontier technologies remain largely inaccessible to women with GDM.

To address this gap, it is essential to integrate broader pregnancy progression management into a condition-specific management app that requires thoughtful design to ensure relevance across different stages of pregnancy and to balance general maternal health management with condition-focused support. As recommended by the 2015 NICE guidance, pregnancy progression management is an important component of GDM management [37]. This requires additional attention on fetal growth and well-being, indicating that additional screening test results and fetal self-observation data recording should be considered [37]. Additional evidence found that women with GDM preferred mHealth technologies that were closely aligned with their pregnancy needs, such as reminders for antenatal visits and pregnancy stages, and they particularly valued educational information related to their pregnancies [16,46].

The findings emphasize the potential of digital tools to provide support for individualized care. It calls for greater integration of automated technologies into app design. This requires co-design approaches involving women and health care professionals to ensure that future apps move from basic features to personalized, interactive, and context-based support for GDM management. Additionally, further exploration is needed to evaluate the automated app feature designs regarding their usability, effectiveness, and safety in real-world contexts.

### Recommendations for Development

Given the wide variation in features and techniques available in current GDM apps, and the range of factors that may influence app development, we adopted an evidence-based approach to summarize broadly applicable recommendations for the development of apps for women with GDM (Textbox 1). These recommendations outline options for core functional design, while also identifying optional advanced features that may be incorporated where appropriate.

Textbox 1. **App development recommendations.**

**Education**

Information about gestational diabetes (GDM):

- Include information on the mechanism of GDM, its relevant risks, and impact on pregnancy. The information should aim to help women recognize the importance of monitoring their blood glucose levels.

Dietary management information:

- The rationale for dietary management and basic dietary strategies should be explained, with links to practical and reliable resources, such as guidance on low-glycemic index food choices.

Physical activity information:

- Provide information about the appropriate types of exercise for pregnant women, optimal timing for exercise, the importance of physical activity, and considerations for ensuring safety.
- Information should be provided on appropriate exercise duration and intensity, conditions in which exercise is not suitable, and when exercise should be stopped if certain symptoms occur.

Self-monitoring skills:

- Provide information about how to perform self-monitoring, including capillary blood glucose testing skills, appropriate timing of testing (for example, fasting, before or after meals, and before bedtime), and evidence-based target ranges for blood glucose levels.

Pregnancy-relevant information:

- Provide information on pregnancy progression, including the different stages of pregnancy and corresponding information about the baby, such as fetal growth.

Basic techniques:

- Text information delivery.

Advanced techniques:

- Graphs, pictures, and videos in information delivery.

Development considerations:

- The primary aim of providing information is to increase women’s awareness of self-monitoring and, in turn, improve their skills. Information should be clear, easy to understand, and sufficient to support understanding.
- Aside from basic information, consider practical resources, such as food recipes or exercise tutorial videos, that support behavior changes.
- Resources provided should be evidence-based and reliable.

**Data management**

Types of data:

- Ensure blood glucose data is accurately recorded and clearly visualized in the app. Consider other essential data, including dietary intake, physical activity, symptoms, medication usage, fetal growth, and fetal well-being, to be recorded within the app.

Basic techniques:

- Manual data recording using text, data visualization using tables, and initial data evaluation by preset normal values.

Advanced techniques:

- Automated data transmission (eg, Bluetooth and image recognition techniques), graphic data visualization to show trends, combination charts to cross-check different types of data, and personalized suggestions based on recorded data.

Development considerations:

- Enable easy data recording, prioritizing automated digital techniques over manual text entry, and provide appropriate indicators and options to improve efficiency.
- Considering digital data analysis techniques to evaluate patients’ recorded data, and where appropriate, to offer initial suggestions to support patients.

**Communication**

Features:

- Communication with health care professionals and peers.

Basic techniques:

- Easy-access contact buttons to start phone calls or send text messages.

Advanced techniques:

- Built-in chat box, online forum.

Development considerations:

- Ensure access to health care professionals within the app.
- The features should be designed to help address the women’s questions in real time.
- Specific staffing roles should be considered to support and manage communication modules within the app.

**Coaching, customization, and technical issues**

Reminding and adherence:

- Basic reminders should be provided for blood glucose testing and other relevant activities.
- More advanced features may include reminders when tests are missed, motivational messages to acknowledge achievements, and rewards linked to milestones.
- Development considerations: the aim of developing such features should be to enable dynamic and intelligent tracking of women’s behaviors, using reminders and notifications to help prevent unhealthy behaviors, support lifestyle adjustment, and encourage women’s behavior change.

Customization:

- Recommended features: demographic data recording, personalized measurement units, customizable normal data ranges, multilanguage options, customizable self-monitoring goals, and a customizable data visualization dashboard.
- Development considerations: the diverse needs, demographic background, and levels of literacy of the women should be considered in app development.

Technical issues:

- Ensure the data details are accurately captured in the app, including numerical precision (for example, decimals), measurement units, and time of data entry.
- Ensure data visualizations are clear and well-organized, and that every recorded data point can be easily viewed.
- Minimize or eliminate connectivity issues and app crashes.

### Strengths and Limitations

The strengths of this review include its standardized process and comprehensiveness of evaluation. Standardized evaluation tools were used to measure the quality and multifunctionality of mHealth apps for GDM. In addition, it extends the TECH framework by Gasteiger et al [26] by introducing a more context-based, user-informed, and evidence-informed approach, integrating BCTs, NICE recommendations, and women’s preferences, enabling a detailed evaluation of app multifunctionality.

However, several limitations should be considered when interpreting the findings. First, the search was conducted only once on a single day. While this minimizes duplication of included apps, it may have resulted in an incomplete capture of all available apps due to the dynamic nature of app store listings, which can be influenced by advertising and other factors. Second, the decision to restrict the search to UK app stores, while ensuring consistency with UK-based NICE guidelines, may have limited the scope and breadth of the review by excluding insights from apps available in other regions. Third, our findings reflect the status of apps in June 2024. Given the high turnover and frequent updates of apps, their scores, features, and technical issues may have changed postreview, which may affect reproducibility.

### Conclusion

This study explored the quality and multifunctionality of commercially available apps for GDM self-management, focusing on the cash-free features, following the TECH and PRISMA frameworks. Our findings highlight that GDM apps should provide more interactive features and support comprehensive pregnancy care, rather than being limited to glucose management and using basic digital techniques. Valuable insights can be drawn from other models of remote pregnancy care, especially those implemented under unavoidable circumstances, such as in rural settings or during the COVID-19 pandemic, where integration of data was required to collect from remote monitoring approaches [47,48]. On one hand, when designing digital technologies for a specific pregnancy complication, it is important to integrate general pregnancy-related data, such as self-reported symptoms, so that they can be managed alongside the target condition [47]. On the other hand, efforts should be made to enable the in-person services and advice to be available online, ensuring that women receive adequate support while engaging in self-management [48]. Taken together, the evidence highlights an emerging research and design agenda for future mHealth app design—one that prioritizes the integration of comprehensive pregnancy progression management and data interconnectivity to better meet the complex requirements of GDM management.

## Supplementary material

10.2196/76862 Multimedia Appendix 1 Eligibility criteria guided by the TECH framework. TECH: target user, evaluation focus, connectedness, and health domain.

10.2196/76862 Multimedia Appendix 2 Evidence of evaluation lists.

10.2196/76862 Multimedia Appendix 3 Enlarged PRISMA diagram. PRISMA: Preferred Reporting Items for Systematic Reviews and Meta-Analyses.

10.2196/76862 Multimedia Appendix 4 Quality appraisal.

10.2196/76862 Multimedia Appendix 5 GDM-adapted features and techniques. GDM: gestational diabetes.

10.2196/76862 Multimedia Appendix 6 General features.

## Abbreviations

BCT: behavior change technique
GDM: gestational diabetes
ICC: interclass correlation coefficient
IMS: The IMS Institute for Healthcare Informatics Functionality Score
MARS: Mobile App Rating Scale
mHealth: mobile health
NICE: National Institute for Health and Care Excellence
PRISMA: Preferred Reporting Items for Systematic Reviews and Meta-Analyses
TECH: target user, evaluation focus, connectedness, health domain
