# Supplementary material for: Quality and Multifunctionality in Mobile Apps for Gestational Diabetes: Systematic App Review
Source: JMIR Mhealth Uhealth. 2026 Feb 5;14:e76862. doi: 10.2196/76862 (PMC12875605; doi:10.2196/76862)
Supplement: Multimedia Appendix 1 — Eligibility criteria guided by the TECH framework. TECH: target user, evaluation focus, connectedness, and health domain. [file mhealth-v14-e76862-s001.docx]

| **Acronym** | **Inclusion Criteria** | **Examples** |
| --- | --- | --- |
| T (Target user) | Women with GDM | Inclusion:   - Mobile applications targeted for women with gestational diabetes. - Mobile applications targeted for pregnancy use but with GDM components. - Mobile applications targeted for diabetes users but claim to be suitable for gestational diabetes in description.   Exclusion:   - Mobile applications tailored for pregnancy users without gestational diabetes components. - Mobile applications tailored for diabetes users without referring to the gestational diabetes population in description. - Mobile applications tailored for healthcare professional training and education. |
| E (Evaluation focus) | App characteristics, quality, functionality | Content of information:   - Types of information   Functionality and quality:   - Types of feature - Quality of features |
| C (Connectedness) | App with external devices | External devices include:   - Glucose meter with or without Bluetooth connection to the app - Wearable smartwatch for pedometer |
| H (Health domain) | Mobile applications suitable for antenatal care | Inclusion:   - Mobile applications suitable for antenatal care, including relevant features such as data recording, behavioural change features to improve blood glucose control, symptoms recording, and help seeking.   Exclusion:   - Mobile applications which primarily focus on the prevention or postpartum care of GDM patients. |
