# Supplementary material for: Quality and Multifunctionality in Mobile Apps for Gestational Diabetes: Systematic App Review
Source: JMIR Mhealth Uhealth. 2026 Feb 5;14:e76862. doi: 10.2196/76862 (PMC12875605; doi:10.2196/76862)
Supplement: Multimedia Appendix 3 — Enlarged PRISMA diagram. PRISMA: Preferred Reporting Items for Systematic Reviews and Meta-Analyses. [file mhealth-v14-e76862-s003.pdf]

PRISMA diagram

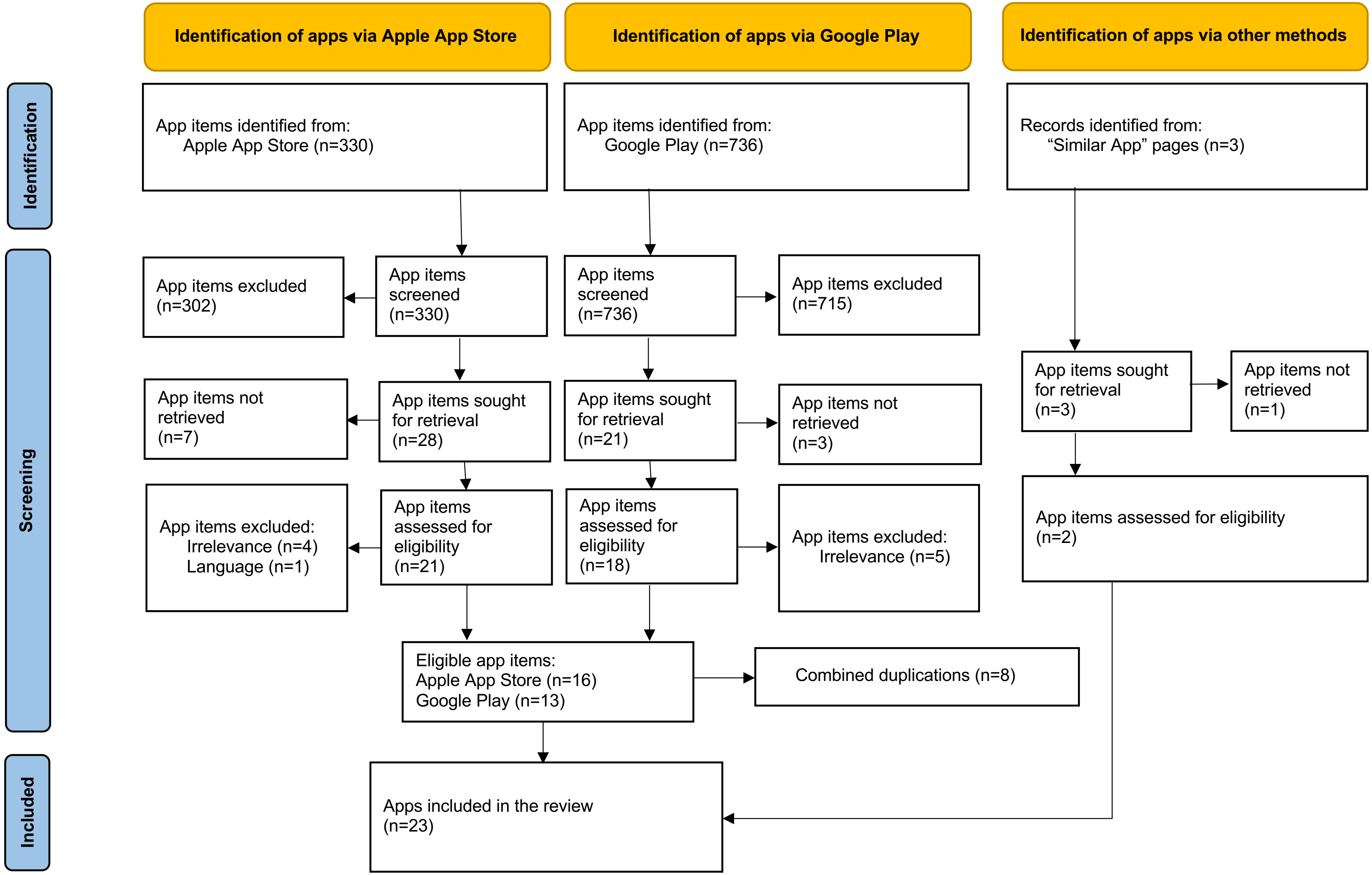

From: Page MJ, McKenzie JE, Bossuyt PM, Boutron I, Hoffmann TC, Mulrow CD, et al. The PRISMA 2020 statement: an updated guideline for reporting systematic reviews. BMJ 2021;372:n71. doi: 10.1136/bmj.n71.
